# Association Between Uric Acid‐to‐HDL Cholesterol Ratio and Sarcopenia: Sex‐Specific Patterns From NHANES 2011–2018

**DOI:** 10.1155/ije/9746996

**Published:** 2026-05-11

**Authors:** Tingting Liu, Xi Liu, Jianling Fan, Hao Chen, Ping Chen, Qiong Xu, Tao Huang, Jiaoyang Zheng

**Affiliations:** ^1^ Department of Health Management Center, The Second Affiliated Hospital of Naval Medical University, Shanghai, China; ^2^ Department of Urology, Ruijin Hospital Lu Wan Branch, Shanghai Jiao Tong University School of Medicine, Shanghai, China, shsmu.edu.cn

**Keywords:** gender differences, NHANES, restricted cubic spline, sarcopenia, UHR

## Abstract

**Objective:**

Sarcopenia, characterized by progressive skeletal muscle mass and strength loss, significantly impacts elderly health. The uric acid‐to‐high‐density lipoprotein cholesterol Ratio (UHR), an emerging metabolic‐inflammatory marker, may be associated with sarcopenia, but large‐scale studies are lacking.

**Methods:**

We conducted a cross‐sectional analysis using NHANES 2011‐2018 data (weighted *N* = 112,108,358). Sarcopenia was defined using FNIH criteria (ASM_BMI_ < 0.512 for women, < 0.789 for men) and UHR calculated as (uric acid/HDL‐C) × 100. Models were adjusted for age, sex, race, education, marital status, poverty‐to‐income ratio, smoking, hypertension, diabetes, total cholesterol, and cancer history.

**Results:**

After multivariable adjustment, UHR showed significant positive association with sarcopenia (OR = 1.060, 95% CI: 1.041–1.080, *p* < 0.001). The highest UHR tertile demonstrated 124.4% higher odds compared to the lowest (OR = 2.244, 95% CI: 1.713–2.940, *p* < 0.001). Sex‐stratified analysis revealed distinct patterns: males exhibited monotonically increasing odds with higher UHR, while females displayed an inverted U‐shaped relationship with an inflection point at UHR = 11.111.

**Conclusion:**

Elevated UHR significantly associates with sarcopenia with pronounced gender differences and may represent a promising, cost‐effective biomarker for improved early detection in aging populations.

## 1. Introduction

Sarcopenia, characterized by progressive loss of skeletal muscle mass and strength, is a significant public health concern associated with increased morbidity, mortality, and healthcare costs. In the United States, NHANES data indicate sarcopenia prevalence of 16% in men and 11% in women over 65 years when using Foundation for the National Institutes of Health (FNIH) criteria [1], with associated healthcare expenditures reaching $18.5 billion annually [2]. Sarcopenia not only compromises quality of life but also significantly correlates with increased fall risk, hospitalization rates, and all‐cause mortality, identifying modifiable risk factors is crucial for effective prevention and management. The uric acid‐to‐high‐density lipoprotein cholesterol ratio (UHR), first proposed by Kurtkulagi et al. [3], represents an emerging metabolic‐inflammatory marker that integrates the dual characteristics of uric acid (pro‐inflammatory) and HDL‐C (anti‐inflammatory). Compared to single indices, UHR offers a more comprehensive reflection of metabolic dysregulation and inflammatory imbalance, demonstrating superior performance in risk assessment across multiple chronic conditions.

Recent investigations confirm that UHR independently predicts chronic kidney disease risk (OR: 2.12; 95% CI: 1.92–2.34) [4]; serves as a potent predictor of all‐cause mortality (HR = 1.16, 95% CI: 1.05–1.29) and cardiovascular mortality (HR = 1.2, 95% CI: 1–1.45) [5]; and accurately assesses insulin resistance in diabetic patients (AUC = 0.665 for males and 0.717 for females, all *p* < 0.01) [6]. These findings establish UHR’s significant value in evaluating chronic inflammation and metabolic dysfunction. NHANES‐based investigations have begun exploring UHR’s utility in U.S. epidemiologic contexts, including analyses in American adults with metabolic liver disease using nationally representative data [7] and long‐term cardiovascular outcomes research supporting UHR as an integrative index [8]. Citing these U.S.‐focused studies strengthens the rationale for evaluating UHR in relation to sarcopenia within American populations.

The theoretical foundation for a potential biological link between sarcopenia and UHR involves three main aspects. Firstly, the pathophysiology of sarcopenia shows significant overlap with the inflammatory and metabolic dysregulation indicated by UHR. Key mechanisms in the development of sarcopenia include chronic low‐grade inflammation, oxidative stress, and insulin resistance [9], all of which are precisely the pathological changes that UHR reflects. Secondly, high uric acid levels can directly cause muscle protein breakdown and mitochondrial dysfunction, while low levels of high‐density lipoprotein cholesterol (HDL‐C) diminish its protective and anti‐inflammatory effects on muscles. Thirdly, there is a strong correlation between UHR and declining kidney function, which is an independent risk factor for sarcopenia [10]. Although this indirect evidence suggests a potential link between UHR and sarcopenia, direct studies investigating this association are lacking, especially large‐scale studies.

Based on our theoretical foundation and identified research gap, we conducted a cross‐sectional study using representative samples from the National Health and Nutrition Examination Survey (NHANES) database to investigate the relationship between UHR and sarcopenia. We hypothesize that higher levels of the UHR are significantly associated with higher odds of sarcopenia, regardless of traditional risk factors. The results may help identify candidate factors associated with sarcopenia for further investigation.

## 2. Materials and Methods

### 2.1. Study Design and Participants

**FIGURE 1:**
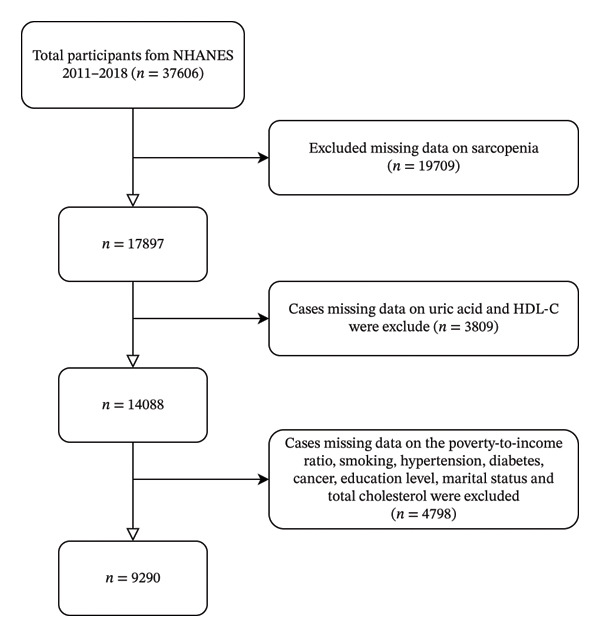
Flowchart of the sample selection from NHANES 2011–2018.

We used data from the NHANES database from four survey cycles (2011–2018) to investigate the correlation between UHR values and sarcopenia in community‐dwelling individuals in the United States using big data analytics. Among the initially recruited participants (*n* = 37,606) from 2011 to 2018, those who lacked data related to sarcopenia (*n* = 19,709) were first excluded. Subsequently, patients who lacked data on uric acid and HDL‐C (*n* = 3809) were excluded. And lastly, we excluded participants with incomplete data on any covariates (*n* = 4798). This exclusion included missing information on factors such as poverty‐to‐income ratio (PIR), smoking status, hypertension, diabetes, cancer, education level, marital status, and total cholesterol (TC). After these exclusions, a total of 9290 participants were included in the final analysis (Figure 1). All participants were over 18 years old. The NHANES obtained approval from the Ethics Review Committee of the National Center for Health Statistics (NCHS), and all participants provided informed consent. The de‐identified and anonymous data on the NHANES website (https://www.cdc.gov/nchs/nhanes/) do not require further ethical approval or informed consent for secondary analysis.

### 2.2. Assessment of the UHR

The data utilized for the computation of the UHR were sourced from laboratory findings within the NHANES, specifically identified as “HDL.Doc” for HDL‐C data and “BIOPRO.Doc” for serum uric acid information. The UHR was determined by dividing serum uric acid (mg/dL) by HDL‐C (mg/dL) according to the formula: UHR (%) = (uric acid/HDL‐C) × 100. Participants were then classified into three distinct categories based on their calculated UHR, aligned with UHR tertiles: Tertile 1 (reference group), Tertile 2, and Tertile 3.

### 2.3. Assessment of Sarcopenia

The regional lean mass of participants was measured using dual‐energy X‐ray absorptiometry (DXA) scan. Appendicular skeletal muscle mass (ASM) was calculated by summing arms lean mass and legs lean mass. BMI (measured as a person’s weight in kilograms divided by the square of height in meters) was directly obtained from the physical examination reports. According to the guidelines established by FNIH, individuals in the NHANES database are diagnosed with sarcopenia based on an adjusted skeletal muscle mass index (ASM_BMI_) of < 0.512 for women and < 0.789 for men. In this study, sarcopenia was defined using DXA‐derived muscle mass indices according to FNIH cutoffs, without direct assessment of muscle strength or physical performance.

### 2.4. Covariates

To mitigate the influence of potential confounding variables, we incorporated a diverse array of covariates pertinent to the UHR and sarcopenia, which encompassed sociodemographic factors, lifestyle habits, and health metrics. The sociodemographic factors comprised age, sex, ethnicity, education level, marital status, and PIR. To evaluate lifestyle habits, we posed a singular question to participants regarding smoking: “Have you smoked more than 100 cigarettes in your lifetime?” They were permitted to respond with either “yes” or “no.” Health status was determined by a self‐reported questionnaire that documented the history of hypertension, diabetes, and cancer. Data concerning TC were acquired from laboratory records labeled “TCHOL.Doc.”

### 2.5. Statistical Analysis

The dataset underwent weighting in accordance with the established sample weighting protocols of NHANES to maintain the representativeness of the sample. We constructed three distinct models to account for potential confounding variables and employed weighted multivariable regression analyses to evaluate the relationships between UHR and the incidence of sarcopenia. Model 1 did not incorporate any adjustments for covariates. In Model 2, adjustments were made for age, sex, race, education level, marital status, and PIR. Model 3 is the fully adjusted model and includes additional adjustments for smoking status, hypertension, diabetes, TC levels, and cancer history. Furthermore, we utilized restricted cubic spline (RCS) models to explore the nonlinear dose–response relationship between markers derived from peripheral blood and the occurrence of sarcopenia, conducting the RCS analysis with three knots positioned at the 5th, 50th, and 95th percentiles. In addition, SHAP (SHapley Additive exPlanations) analysis and Boruta feature filtering were performed as exploratory complementary approaches to assess variable importance and model interpretability.

To ensure the robustness of our findings across diverse sample characteristics, subgroup analyses were carried out based on sex, race, marital status, education level, smoking status, hypertension, diabetes, and cancer. Generalized variance inflation factor (GVIF) values were computed to assess collinearity, with a GVIF of less than 5 indicating an absence of collinearity. Data processing was executed utilizing R software version 4.1.0 (R Core Team, Vienna, Austria) and DecisionLinnc1.0 software (https://www.statsape.com). All *p* values were two‐sided, with a *p* value of less than 0.05 considered to indicate statistical significance.

## 3. Results

### 3.1. Demographic and Clinical Characteristics of Participants With and Without Sarcopenia

Out of 112,108,358 participants (weighted), 7,897,904 (7.05%) had sarcopenia. Participants with sarcopenia were significantly older (43.05 ± 11.84 vs. 39.03 ± 11.59 years, *p* < 0.001) and had lower family income‐to‐poverty ratios (2.44 ± 1.59 vs. 2.99 ± 1.67, *p* < 0.001). The UHR was significantly higher in the sarcopenia group (12.84 ± 5.72 vs. 11.02 ± 4.95, *p* < 0.001), while TC levels showed no significant difference (*p* = 0.066). Comorbidity analysis revealed higher prevalence of hypertension (29.79% vs. 21.39%, *p* < 0.001), diabetes (15.50% vs. 4.99%, *p* < 0.001), and cancer (7.35% vs. 4.58%, *p* = 0.032) in the sarcopenia group. Smoking status was comparable between groups (*p* = 0.434). Significant racial disparities were observed (*p* < 0.001), as Mexican Americans exhibited the highest prevalence of sarcopenia at 25.56%, whereas non‐Hispanic Blacks had the lowest prevalence at 3.57%. Educational attainment was inversely associated with sarcopenia (*p* < 0.001), with lower representation of college graduates (18.61% vs. 33.86%) and higher proportion of individuals with less than 9th grade education (11.07% vs. 2.97%) in the sarcopenia group. Gender distribution (*p* = 0.087) and marital status (*p* = 0.170) showed no significant differences between groups. These demographic characteristics and clinical findings are presented in Table 1, suggesting that sarcopenia is associated with older age, metabolic abnormalities, chronic conditions, lower socioeconomic status, and varies significantly across racial and educational groups. We performed GVIF analysis to evaluate multicollinearity in our regression model (Table S1), finding all variables below GVIF 2.0 and adjusted GVIF 1.25, indicating no serious multicollinearity. UHR had a GVIF of 1.31 (adjusted 1.14), well below the 5.0 threshold, suggesting mild correlation. Age’s GVIF was 1.51, while education (1.65), race (1.38), and marital status (1.45) also showed low multicollinearity, with adjusted values of 1.06, 1.04, and 1.04, respectively. These results enhance our confidence in the regression estimates, allowing reliable assessment of UHR’s relationship with sarcopenia.

**TABLE 1:** Baseline characteristics of study participants by sarcopenia status (weighted).

Characteristic	Overall (*N* = 112,108,358)	Nonsarcopenia (*n* = 104,210,454)	Sarcopenia (*n* = 7,897,904)	*p* value
*Demographic characteristics*				
Age (years), mean ± SD	39.31 ± 11.66	39.03 ± 11.59	43.05 ± 11.84	< 0.001
Sex, *n* (%)				0.087
Female	55,946,062 (49.90)	52,321,379 (50.21)	3,624,683 (45.89)	
Male	56,162,296 (50.10)	51,889,075 (49.79)	4,273,220 (54.11)	
Race/ethnicity, *n* (%)				< 0.001
Non‐Hispanic White	70,234,466 (62.65)	66,379,233 (63.70)	3,855,233 (48.81)	
Non‐Hispanic Black	11,962,543 (10.67)	11,680,902 (11.21)	281,641 (3.57)	
Mexican American	11,340,417 (10.12)	9,321,666 (8.95)	2,018,751 (25.56)	
Other Hispanic	7,905,167 (7.05)	6,965,337 (6.68)	939,830 (11.90)	
Other race	10,665,765 (9.51)	9,863,317 (9.46)	802,448 (10.16)	

*Socioeconomic status*				
Family income‐to‐poverty ratio, mean ± SD	2.95 ± 1.67	2.99 ± 1.67	2.44 ± 1.59	< 0.001
Education level, *n* (%)				< 0.001
Less than 9th grade	3,973,383 (3.54)	3,098,724 (2.97)	874,659 (11.07)	
9–11th grade	9,679,580 (8.63)	8,783,628 (8.43)	895,951 (11.34)	
High school graduate	24,190,370 (21.58)	21,926,833 (21.04)	2,263,537 (28.66)	
Some college or AA degree	37,510,488 (33.46)	35,116,421 (33.70)	2,394,067 (30.31)	
College graduate or above	36,754,537 (32.78)	35,284,847 (33.86)	1,469,690 (18.61)	
Marital status, *n* (%)				0.170
Married	57,986,429 (51.72)	53,833,832 (51.66)	4,152,597 (52.58)	
Never married	27,390,507 (24.43)	25,728,328 (24.69)	1,662,179 (21.05)	
Living with partner	11,771,389 (10.50)	11,014,915 (10.57)	756,475 (9.58)	
Divorced	10,536,330 (9.40)	9,645,506 (9.26)	890,825 (11.28)	
Separated	3,068,696 (2.74)	2,708,689 (2.60)	360,007 (4.56)	
Widowed	1,355,006 (1.21)	1,279,185 (1.23)	75,821 (0.96)	

*Clinical characteristics*				
UHR, mean ± SD	11.14 ± 5.03	11.02 ± 4.95	12.84 ± 5.72	< 0.001
Total cholesterol (mg/dL), mean ± SD	191.53 ± 40.26	191.22 ± 40.07	195.59 ± 42.56	0.066
Hypertension, *n* (%)	24,645,705 (21.98)	22,292,552 (21.39)	2,353,153 (29.79)	< 0.001
Diabetes, *n* (%)	6,424,631 (5.73)	5,200,642 (4.99)	1,223,989 (15.50)	< 0.001
Cancer, *n* (%)	5,357,919 (4.78)	4,777,110 (4.58)	580,808 (7.35)	0.032
Smoking, *n* (%)	46,102,987 (41.12)	43,002,461 (41.27)	3,100,526 (39.26)	0.434

*Note:* *p* values were derived from *t*‐tests for continuous variables and chi‐square tests for categorical variables. Percentages may not sum to 100% due to rounding.

Abbreviations: SD, standard deviation; UHR, uric acid‐to‐high‐density lipoprotein cholesterol ratio.

### 3.2. Association Between UHR and Sarcopenia

Table 2 presents the association between the UHR and sarcopenia. In the unadjusted model (Model 1), each unit increase in UHR was associated with 6.3% higher odds of sarcopenia (OR = 1.063, 95% CI: 1.047–1.080, *p* < 0.001). The association remained significant after adjusting for age, sex, race, education level, marital status, and PIR (Model 2), showing a slightly stronger effect size (OR = 1.067, 95% CI: 1.048–1.086, *p* < 0.001). Further adjustment for smoking, hypertension, diabetes, TC, and cancer (Model 3) confirmed that the association between UHR and sarcopenia remained significant (OR = 1.060, 95% CI: 1.041–1.080, *p* < 0.001). To explore potential nonlinear relationships between UHR and sarcopenia, we categorized UHR into tertiles: Q1, Q2, and Q3. Compared to the lowest tertile (Q1), the middle tertile (Q2) showed significantly higher odds of sarcopenia, with a 63.0% increase in the unadjusted model (OR = 1.630, 95% CI: 1.274–2.084, *p* < 0.001) and a 53.6% increase in the fully adjusted model (OR = 1.536, 95% CI: 1.200–1.967, *p* = 0.001). The highest tertile (Q3) demonstrated even more pronounced associations, with a 137.7% increase in the unadjusted model (OR = 2.377, 95% CI: 1.842–3.067, *p* < 0.001) and a 124.4% increase in the fully adjusted model (OR = 2.244, 95% CI: 1.713–2.940, *p* < 0.001). The trend test showed a significant increase in sarcopenia odds as UHR levels rose (*p* for trend < 0.001).

**TABLE 2:** Relationship between UHR and sarcopenia.

UHR	Model 1		Model 2		Model 3	
	OR (95% CI)	*p* value	OR (95% CI)	*p* value	OR (95% CI)	*p* value
UHR	1.063 (1.047, 1.080)	< 0.001	1.067 (1.048, 1.086)	< 0.001	1.060 (1.041, 1.080)	< 0.001
UHR (Tertile)						
Q1	Reference		Reference		Reference	
Q2	1.630 (1.274, 2.084)	< 0.001	1.615 (1.260, 2.069)	< 0.001	1.536 (1.200, 1.967)	0.001
Q3	2.377 (1.842, 3.067)	< 0.001	2.448 (1.875, 3.198)	< 0.001	2.244 (1.713, 2.940)	< 0.001
*p* for trend	< 0.001		< 0.001		< 0.001	

*Note:* Model 1: Unadjusted model; Model 2: Adjusted for age, sex, race, education level, marital status, and poverty‐to‐income ratio; Model 3: Additionally adjusted for smoking, hypertension, diabetes, total cholesterol, and cancer.

Abbreviation: CI, confidence interval.

These findings indicate an independent and robust positive association between UHR and sarcopenia, with a clear dose–response pattern. UHR, as a simple biochemical indicator, may have important clinical value in risk assessment for sarcopenia, as the association remains significant even after accounting for multiple traditional risk factors. This suggests that UHR may reflect a state of metabolic dysregulation associated with sarcopenia; however, the cross‐sectional design does not allow conclusions regarding temporality or causality.

### 3.3. RCS Analysis

Weighted RCS analysis was used to model and visualize the relationship between peripheral blood–derived markers and the odds of sarcopenia. Our RCS analysis revealed a significant nonlinear association between UHR and the odds of sarcopenia. As illustrated, UHR demonstrated a significant nonlinear dose–response relationship with sarcopenia odds across three adjustment models (*p* for overall association < 0.001 in all models). In the unadjusted model (Figure 2(a)), sarcopenia odds gradually increased with rising UHR values, with an accelerated increase observed when UHR exceeded approximately 10, reaching maximum effect at higher UHR values (> 30). This nonlinear pattern remained consistent after adjustment for demographic factors (Figure 2(b)) and further adjustment for health‐related conditions (Figure 2(c)), indicating that the association between UHR and sarcopenia was independent of known confounding factors.

FIGURE 2Nonlinear associations between uric acid‐to‐high‐density lipoprotein cholesterol ratio (UHR) and sarcopenia. (a) Unadjusted model showing significant nonlinearity. (b) Model adjusted for age, sex, race, education level, marital status, and poverty‐to‐income ratio. (c) Fully adjusted model with additional adjustment for smoking status, hypertension, diabetes, total cholesterol, and cancer history. Sex‐stratified analyses in (d) males showing monotonically increasing relationship and (e) females demonstrating an inverted U‐shaped association. The solid line represents the estimated odds ratios, and the shaded area indicates the 95% confidence interval. The horizontal dashed line indicates an odds ratio of 1.0 (reference).

**Figure:**
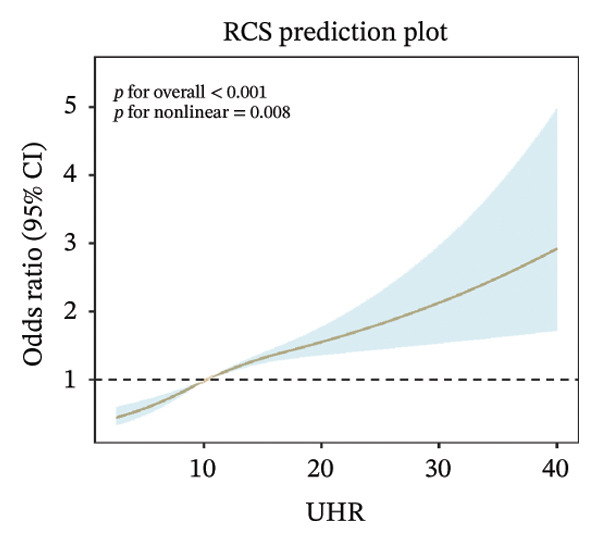
(a)

**Figure:**
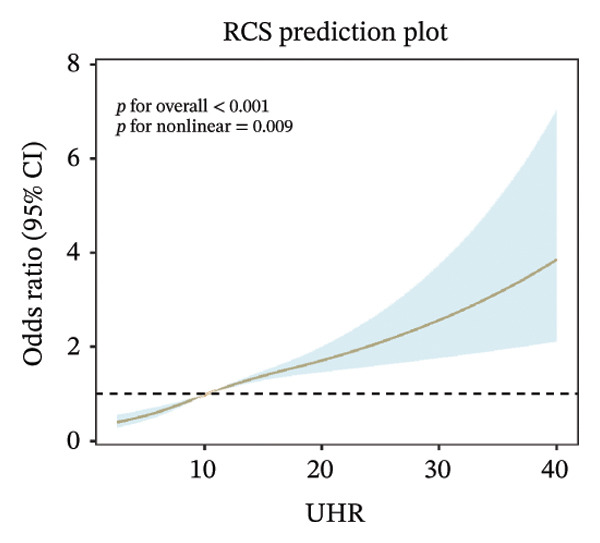
(b)

**Figure:**
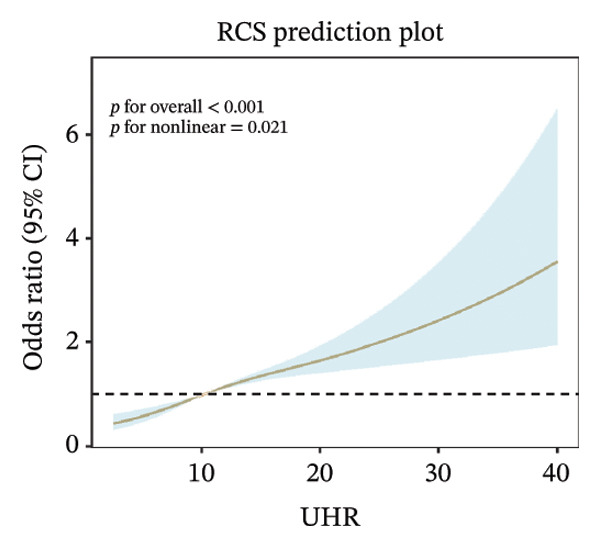
(c)

**Figure:**
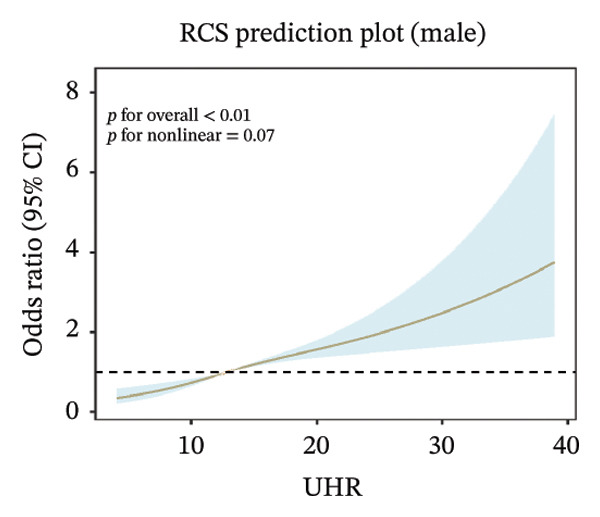
(d)

**Figure:**
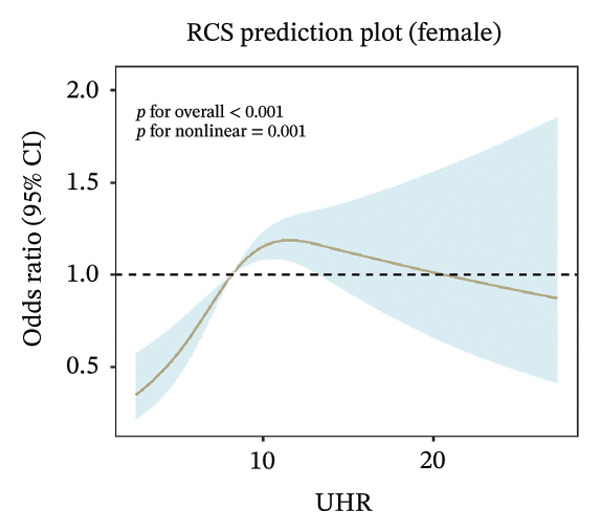
(e)

Sex‐stratified analysis revealed notable gender differences. In males (Figure 2(d)), UHR demonstrated a monotonically increasing nonlinear relationship with sarcopenia odds (*p* for overall association < 0.001, *p* for linearity = 0.07), with odds continuously rising as UHR increased, particularly pronounced after UHR > 15. In contrast, females (Figure 2(e)) exhibited a distinctly different pattern: an inverted U‐shaped relationship between UHR and sarcopenia odds (*p* for overall association < 0.001, *p* for linearity = 0.001), with odds peaking at UHR approximately 10, followed by a gradual decline as UHR continued to increase. This difference between sexes suggests that UHR may have distinct roles in the pathophysiological mechanisms of sarcopenia in males compared to females.

### 3.4. Sex‐Specific Threshold Effect of UHR on Sarcopenia in Females

We conducted threshold effect analysis to examine the potential nonlinear relationship between UHR and sarcopenia specifically in the female population. Our analysis revealed a significant sex‐specific threshold effect of UHR on sarcopenia odds in women (Table 3). In the linear regression model for females, UHR demonstrated a significant positive association with sarcopenia (OR = 1.039, 95% CI: 1.011–1.068, *p* = 0.0056), suggesting that each unit increase in UHR corresponded to 3.9% higher odds of sarcopenia when modeled as a simple linear relationship. However, the threshold analysis identified a significant inflection point at UHR = 11.111, where the relationship between UHR and sarcopenia markedly changed in women. Below this threshold, the association between UHR and sarcopenia was substantially stronger than suggested by the linear model, with each unit increase in UHR associated with 13.7% higher odds of sarcopenia in females (OR = 1.137, 95% CI: 1.075–1.204, *p* < 0.0001). In contrast, above the threshold of 11.111, the association was no longer statistically significant and suggested a possible inverse relationship in women (OR = 0.963, 95% CI: 0.911–1.013, *p* = 0.1616). This finding is consistent with the inverted U‐shaped relationship observed in our RCS analysis for females. The log‐likelihood ratio test yielded a *p* value of 0.0002, providing strong statistical evidence that the threshold model significantly improved the fit compared to the linear model in the female cohort. This finding confirms that the relationship between UHR and sarcopenia in women is not simply linear but exhibits a clear threshold effect, with UHR being a significant associated factor primarily when below 11.111.

**TABLE 3:** The results of two‐piecewise linear regression model for the association between UHR and sarcopenia in females.

Inflection point of UHR	Effect size (OR)	95% CI	*p* value
< 11.111	1.137	1.075 to 1.204	< 0.001
≥ 11.111	0.963	0.911 to 1.013	0.162

*Note:* Effect: Sarcopenia cause: UHR. Log‐likelihood ratio test for nonlinearity: *p* = 0.0002. Linear model effect size (OR): 1.039 (95% CI: 1.011–1.068, *p* = 0.0056).

### 3.5. Subgroup Analysis of the Association Between UHR and Sarcopenia

**FIGURE 3:**
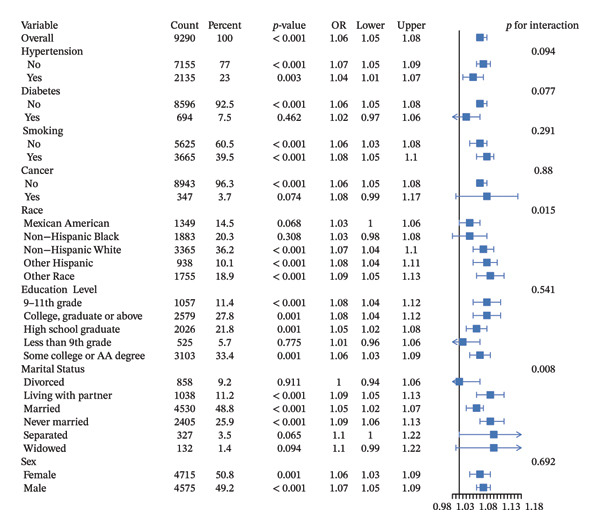
Subgroup analyses of the association between UHR and sarcopenia. UHR, uric acid‐to‐high‐density lipoprotein cholesterol ratio; OR, odds ratio; CI, confidence interval.

To examine whether the relationship between UHR and sarcopenia varied across population subgroups, we conducted comprehensive stratified analyses (Figure 3). In the overall population (*n* = 9290), each unit increase in UHR was associated with 6% higher odds of sarcopenia (OR = 1.06, 95% CI: 1.05–1.08, *p* < 0.001). Our analyses revealed significant effect modification by race (*p* for interaction = 0.015) and marital status (*p* for interaction = 0.008). The association between UHR and sarcopenia varied substantially across racial groups: it was strongest in the “Other Race” category (OR = 1.09, 95% CI: 1.05–1.13, *p* < 0.001) and “Other Hispanic” participants (OR = 1.08, 95% CI: 1.04–1.11, *p* < 0.001), moderate in non‐Hispanic Whites (OR = 1.07, 95% CI: 1.04–1.10, *p* < 0.001), and did not reach statistical significance in Mexican Americans (OR = 1.03, 95% CI: 1.00–1.06, *p* = 0.068) and non‐Hispanic Blacks (OR = 1.03, 95% CI: 0.98–1.08, *p* = 0.308). These racial differences may reflect the influence of genetic, environmental, or socioeconomic factors that modify how UHR impacts muscle health. Marital status also significantly modified the UHR‐sarcopenia relationship. The association was most pronounced among separated individuals (OR = 1.10, 95% CI: 1.00–1.22, *p* = 0.065), widowed individuals (OR = 1.10, 95% CI: 0.99–1.22, *p* = 0.094), never married individuals (OR = 1.09, 95% CI: 1.06–1.13, *p* < 0.001), and those living with partners (OR = 1.09, 95% CI: 1.05–1.13, *p* < 0.001). This pattern suggests that social factors, lifestyle behaviors, or psychological aspects associated with different marital statuses may influence the impact of UHR on muscle health. We observed marginally significant interactions with cardiometabolic conditions. The UHR–sarcopenia association was slightly stronger in individuals without hypertension (OR = 1.07, 95% CI: 1.05–1.09, *p* < 0.001) compared to those with hypertension (OR = 1.04, 95% CI: 1.01–1.07, *p* = 0.003; *p* for interaction = 0.094). Similarly, while a significant association was found in nondiabetic participants (OR = 1.06, 95% CI: 1.05–1.08, *p* < 0.001), it was attenuated and not statistically significant in those with diabetes (OR = 1.02, 95% CI: 0.97–1.06, *p* = 0.462; *p* for interaction = 0.077). These findings suggest that metabolic disorders may potentially modify the pathophysiological pathways linking UHR and sarcopenia, although these interactions did not reach conventional statistical significance. No significant interactions were observed across smoking status (*p* for interaction = 0.291), cancer history (*p* for interaction = 0.880), education level (*p* for interaction = 0.541), or sex (*p* for interaction = 0.692). Both females (OR = 1.06, 95% CI: 1.03–1.09, *p* = 0.001) and males (OR = 1.07, 95% CI: 1.05–1.09, *p* < 0.001) showed similar associations, although the RCS analysis suggested different underlying patterns between sexes.

### 3.6. Exploratory Feature Importance Analyses Using SHAP and Boruta Methodologies

To complement the primary regression analyses, we employed two complementary feature importance methodologies: SHAP (SHapley Additive exPlanations) analysis and Boruta feature filtering to evaluate the relative importance of variables associated with sarcopenia. The SHAP importance analysis (Figure 4(a)) revealed that educational attainment was the predominant predictor (SHAP value 0.12), substantially outweighing other variables. This finding highlights the critical role of socioeconomic determinants in sarcopenia risk, likely mediated through multiple pathways including health literacy, lifestyle behaviors, nutritional quality, and preventive healthcare utilization. Hypertension emerged as the second most influential predictor (SHAP value 0.045), followed by age (SHAP value 0.04), suggesting that cardiovascular health may have comparable importance to chronological aging in determining muscle integrity. The UHR, our biomarker of interest, demonstrated meaningful importance (SHAP value 0.025), supporting its potential clinical utility in sarcopenia risk stratification. Variables including diabetes, race, smoking status, and marital status showed progressively diminishing importance, while TC, cancer history, and sex contributed minimally to model predictions.

FIGURE 4Feature importance analysis for sarcopenia prediction using SHAP and Boruta methods. (a) SHAP importance plot showing the relative contribution of each predictor to model predictions. (b) Boruta feature filtering results comparing variable mean importance (red boxes) against reference shadow attributes (green, blue, purple boxes). UHR, uric acid‐to‐high‐density lipoprotein cholesterol ratio; TC, total cholesterol.

**Figure:**
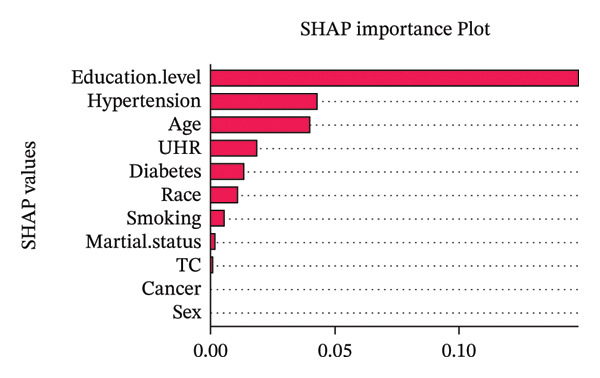
(a)

**Figure:**
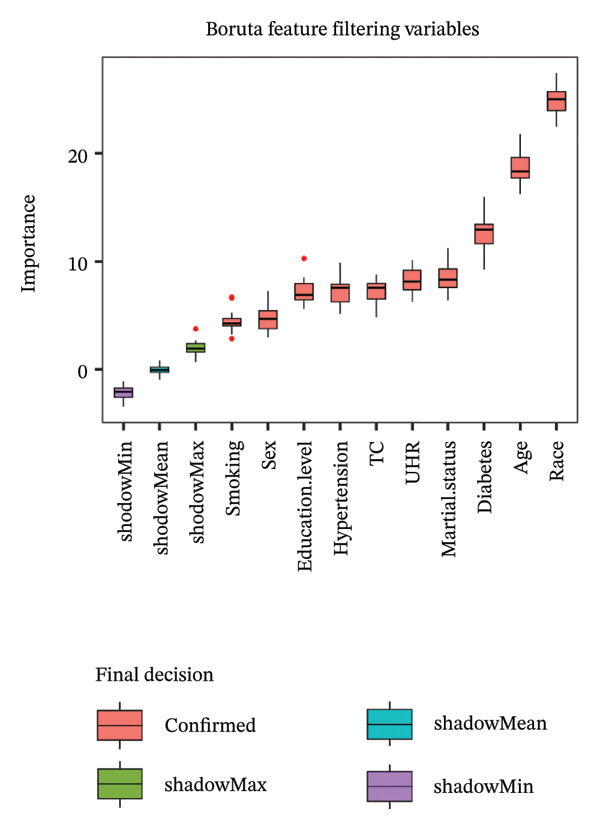
(b)

The Boruta feature filtering algorithm (Figure 4(b)) provided complementary insights through statistical validation of predictor importance. Ten variables achieved confirmed status with importance scores significantly exceeding the reference shadow variables (Table S2), while cancer was rejected (mean importance score = 0.37). Notably, Boruta analysis identified a different importance hierarchy, with race demonstrating the highest statistical significance (mean importance = 24.88, range: 22.44–27.39), followed by age (mean importance = 18.64, range: 16.24–21.77) and diabetes (mean importance = 12.67, range: 9.27–15.95). Marital status (mean importance = 8.46), UHR (mean importance = 8.22), TC (mean importance = 7.25), hypertension (mean importance = 7.23), and education level (mean importance = 7.20) followed in descending order of importance. SHAP values quantify direct impact on prediction magnitude, while Boruta importance scores measure statistical significance relative to random features. Notably, UHR was retained as an important feature across both approaches. Although these analyses do not provide effect estimates or infer independent associations, they offer complementary evidence that UHR remains relevant within a broader multivariable context, thereby supporting the main findings from the regression models.

## 4. Discussion

In this large‐scale cross‐sectional study based on NHANES 2011–2018, we investigated the association between UHR and sarcopenia. Using a representative sample (weighted *N* = 112,108,358 participants), we found that UHR levels were significantly positively associated with sarcopenia odds (OR = 1.060, 95% CI: 1.041–1.080, *p* < 0.001). Compared to the lowest UHR tertile, the highest tertile demonstrated 124.4% higher odds of sarcopenia (OR = 2.244, 95% CI: 1.713–2.940, *p* < 0.001), with a clear dose–response relationship (*p* for trend < 0.001). RCS analysis further revealed a nonlinear association between UHR and sarcopenia, with notable gender differences: a monotonically increasing relationship in males versus an inverted U‐shaped relationship in females, with an inflection point at UHR = 11.111. This study utilized a large sample size and rigorous statistical analyses, providing initial population‐level evidence of an association between UHR and sarcopenia from a metabolic‐inflammation perspective. However, because of the cross‐sectional design, these findings do not establish causality.

Our findings provide evidence of sex‐specific associations between uric acid levels and sarcopenia, extending prior research. Zhou et al. [11] showed that there are significant gender differences in the relationship between uric acid and sarcopenia in Chinese adults. They found protective effects in males, while no significant relationship exists in females. This is similar to our new observation of an inverted U‐shaped relationship in females, indicating essential gender differences in metabolic and muscle health interactions. Lin et al. [12] did not find a bidirectional causal relationship between urate and sarcopenia‐related traits using Mendelian randomization. Conversely, our cross‐sectional analysis of NHANES data (weighted *N* = 112,108,358) reveals more nuanced, nonlinear association patterns that may reconcile these seemingly conflicting results. Our research builds on the findings of Jiang et al. [13], who investigated lipid metabolism indices and sarcopenia in Chinese community‐dwelling individuals but failed to establish a significant HDL‐C–sarcopenia relationship. By examining UHR as a combined metabolic and inflammatory indicator, we discovered complex association patterns with significant gender differences that may more accurately represent the multifactorial causes of sarcopenia. These findings align with Cruz‐Jentoft and Sayer [14] Lancet review identifying inflammation and vascular dysfunction as key pathological mechanisms in sarcopenia development, where HDL‐C likely plays a crucial role through its lipid metabolism regulation and anti‐inflammatory properties.

From a mechanistic perspective, the study by Wang et al. [15] investigated the relationship between UHR and arterial stiffness and revealed significant sex‐specific differences (*p* for interaction = 0.0327). In females, there was a strong positive correlation (*β* = 11.97, 95% CI: 3.79–20.15, *p* = 0.0042), while no significant association was found in males (*β* = 2.34, 95% CI: −1.21–5.89, *p* = 0.1970). These findings underscore the different strengths of the correlations and support our conclusions about gender differences in the UHR–sarcopenia relationship. Huang et al. [16] investigated the relationship between serum lipids and sarcopenia and discovered a nonlinear association between triglycerides and sarcopenia risk. This finding parallels the nonlinear relationship we observed between UHR and sarcopenia, confirming the complex interactions between metabolic factors and muscle health. Additionally, their study indicated that low HDL‐C levels might be associated with increased sarcopenia risk, providing a theoretical basis for our focus on UHR as an integrated indicator. Chung et al.’s [17] meta‐analysis found that in Asian community populations, the prevalence of sarcopenia among diabetic patients was significantly higher than in nondiabetic populations (pooled OR = 1.518, 95% CI = 1.110–2.076, *Z*‐value = 2.611, *p* = 0.009), indicating a close connection between metabolic abnormalities and sarcopenia, providing epidemiological support for our study on the relationship between UHR and sarcopenia. From the perspective of potential biological mechanisms, these gender differences may be related to sex hormone–regulated metabolic and inflammatory pathways, as Morley [18] discussed in his review on hormones and sarcopenia, indicating that estrogen and testosterone exhibit significant differences in regulating muscle protein synthesis and inflammatory responses, which may explain why UHR impacts muscle health differently in males and females. The inverted U‐shaped association observed in women suggests that the relationship between UHR and muscle health may be biologically more complex in females than in males. One possible explanation is that sex‐specific regulation of lipid metabolism, uric acid handling, body fat distribution, and inflammatory responses may modify the association across different UHR ranges. In addition, hormonal status, particularly estrogen‐related metabolic regulation, may partly contribute to this nonlinear pattern. It is also noteworthy that uric acid may exert both antioxidant and pro‐inflammatory effects depending on the physiological context, which could further contribute to nonlinearity.

While UHR demonstrated significant associations with sarcopenia in our analysis, its potential clinical value should be contextualized not only against established inflammatory and metabolic biomarkers but also within emerging multidimensional biomarker research in sarcopenia. Traditional markers such as C‐reactive protein (CRP), neutrophil‐to‐lymphocyte ratio, and individual lipid components have been investigated in sarcopenia contexts with varying degrees of success. UHR’s potential advantage lies in its integration of both pro‐inflammatory (uric acid) and anti‐inflammatory (HDL‐C) components, providing a composite measure of metabolic‐inflammatory status that may offer incremental utility beyond single biomarkers. For instance, in metabolic disorders, UHR has shown better predictive performance compared to individual uric acid or HDL‐C measurements alone [19]. Recent biomarker research in sarcopenia suggests that no single circulating indicator is likely to fully capture the heterogeneous biology of muscle decline. Emerging approaches have expanded beyond conventional inflammatory and metabolic markers to include metabolomic and amino acid–based signatures. For example, urinary amino acid profiling has been proposed as a noninvasive strategy for sarcopenia detection, highlighting the potential relevance of altered amino acid metabolism in muscle loss [20]. In parallel, recent work on inflammation‐related indices in older adults has shown that markers such as UHR may be associated with impaired physical performance, although their discriminative ability appears modest, suggesting that they may be more appropriately interpreted as complementary rather than standalone biomarkers [21]. Therefore, our findings may position UHR not as an isolated marker, but as part of a developing multidimensional biomarker framework for sarcopenia.

Beyond the primary regression models, the SHAP and Boruta analyses were included as complementary exploratory approaches to further assess the potential relevance of UHR in a multivariable setting. While logistic regression quantified the independent association between UHR and sarcopenia, SHAP and Boruta provided additional information on variable importance and model interpretability. The consistent identification of UHR as an important feature across both methods suggests that its relevance was not solely dependent on one modeling strategy, thereby strengthening its potential value as a candidate metabolic‐inflammatory marker in sarcopenia risk profiling.

The potential clinical relevance of this study lies in suggesting that UHR may be a readily available candidate biomarker for sarcopenia risk assessment. Compared to traditional sarcopenia evaluation methods (such as muscle mass measurement and strength testing), UHR detection is cost‐effective and widely applicable across all levels of healthcare facilities, particularly suitable for large‐scale population screening. Additionally, the identified sex‐specific nonlinear association patterns may provide useful hypotheses for future mechanistic and longitudinal studies. If validated in prospective studies, UHR could potentially be considered for incorporation into routine health assessments and sarcopenia risk evaluation frameworks. Furthermore, preventing and delaying sarcopenia development by improving metabolic health indicators provides a novel approach to addressing challenges of population aging. Future research directions include prospective cohort validation of UHR’s predictive value for sarcopenia occurrence and progression, exploration of applicability across different ethnic and regional populations, clarification of the temporal relationship between UHR changes and sarcopenia progression, and in‐depth investigation of molecular mechanisms underlying gender differences, providing a more solid theoretical basis for UHR‐based intervention strategies.

This study has several strengths and innovations. First, our analysis based on NHANES 2011–2018, a nationally representative database, features an extensive sample size (weighted *N* = 112,108,358) with rigorous quality control systems, ensuring reliability and generalizability of results. Second, we innovatively introduced UHR as an integrated metabolic‐inflammatory composite indicator, transcending limitations of traditional single biomarkers to explore potential mechanisms of sarcopenia from the perspective of uric acid and HDL‐C interaction. Regarding data analysis strategies, we employed a multilevel progressive analytical framework: utilizing multivariate logistic regression to examine continuous variable associations, assessing dose‐response relationships through tertile analysis, and exploring nonlinear association patterns using RCS models. Additionally, we conducted comprehensive sex‐stratified analyses, revealing significant gender differences and identifying an inflection point in the UHR–sarcopenia relationship among females (UHR = 11.111). For confounding factor control, we adjusted for a broad set of sociodemographic and clinical covariates, including age, sex, race/ethnicity, education level, marital status, PIR, smoking status, hypertension, diabetes, TC, and cancer history. We also performed a series of sensitivity analyses and subgroup analyses, verifying the robustness of our findings. Through these methodological advantages, we not only established evidence for the association between UHR and sarcopenia but also detailed its nonlinear characteristics and gender differences, providing new perspectives for understanding the pathophysiological mechanisms of sarcopenia.

Despite its numerous strengths, this study has several noteworthy limitations. First, because of the cross‐sectional design, temporal direction and causality between UHR and sarcopenia cannot be established. Second, we used a complete‐case analytic approach, and a substantial number of participants were excluded because of missing DXA, laboratory, or covariate data. This may have introduced selection bias if the included participants differed systematically from those excluded in demographic or health‐related characteristics. Therefore, the representativeness and generalizability of our findings should be interpreted with caution. Third, the outcome was defined solely on the basis of DXA‐derived muscle mass according to the FNIH criteria. Contemporary consensus definitions of sarcopenia also emphasize muscle strength and physical performance. Therefore, our definition may more closely reflect low muscle mass rather than comprehensively defined sarcopenia. Fourth, although we adjusted for multiple sociodemographic and clinical factors, residual confounding from unmeasured variables, including medication use and other lifestyle‐related factors, remains possible. Finally, because NHANES primarily represents the U.S. population, caution is warranted when generalizing these findings to other racial, ethnic, or regional populations. These limitations highlight the need for prospective studies in diverse populations to further validate our findings.

## 5. Conclusion

This large‐scale cross‐sectional study demonstrates significant associations between elevated UHR levels and sarcopenia in U.S. adults, with distinct sex‐specific patterns. However, given the cross‐sectional design, these findings should be interpreted as associative rather than causal. UHR may serve as a candidate metabolic‐inflammatory biomarker for sarcopenia risk assessment and may be best interpreted within a broader multidimensional biomarker framework. Longitudinal studies are needed to clarify temporal relationships and validate its predictive value in diverse populations.

## Author Contributions

Conceptualization and analysis, Tingting Liu and Xi Liu; data curation, Jianling Fan, Hao Chen, and Ping Chen; manuscript edits, Tingting Liu and Qiong Xu; writing–original draft preparation, Xi Liu; writing–review and editing, Jiaoyang Zheng and Tao Huang; supervision, Jiaoyang Zheng; project administration, Jiaoyang Zheng; funding acquisition, Xi Liu.

## Funding

The study was supported by the Shanghai Huangpu District Scientific Research Project Fund (project number HLM202304) and the National High Level Hospital Clinical Research Funding (Grant No. 2022‐PUMCH‐D‐006).

## Disclosure

All authors read and approved the final manuscript.

## Ethics Statement

The NHANES received ethical clearance from the Ethics Review Committee of the National Center for Health Statistics (NCHS) and guaranteed that all participants granted informed consent.

## Consent

Please see the ethics statement.

## Conflicts of Interest

The authors declare no conflicts of interest.

## Supporting Information

Additional supporting information can be found online in the Supporting Information section.

## Supporting information


**Supporting Information** Table S1: Variance Inflation Factor Analysis for Predictor Variables in the Multivariable Model. Table S2: Boruta Feature Filtering Results with Multiple Variables.

## Data Availability

Comprehensive details regarding the NHANES study designs and data are accessible to the public at https://www.cdc.gov/nchs/nhanes/.

## References

[1] Studenski S. A. , Peters K. W. , Alley D. E. et al., The FNIH Sarcopenia Project: Rationale, Study Description, Conference Recommendations, and Final Estimates, Journals of Gerontology: Series A, Biological Sciences and Medical Sciences. (2014) 69, no. 5, 547–558, 10.1093/gerona/glu010, 2-s2.0-84899071546. 24737557 PMC3991146

[2] Janssen I. , Shepard D. S. , Katzmarzyk P. T. , and Roubenoff R. , The Healthcare Costs of Sarcopenia in the United States, Journal of the American Geriatrics Society. (2004) 52, no. 1, 80–85, 10.1111/j.1532-5415.2004.52014.x, 2-s2.0-0346499181. 14687319

[3] Kurtkulagi O. , Tel B. , Kahveci G. et al., Hashimoto’s Thyroiditis is Associated With Elevated Serum Uric Acid to High Density Lipoprotein-Cholesterol Ratio, Romanian Journal of Internal Medicine. (2021) 59, no. 4, 403–408, 10.2478/rjim-2021-0023. 34142519

[4] Cheng Y. , Zhang H. , Zheng H. et al., Association Between Serum Uric Acid/HDL-Cholesterol Ratio and Chronic Kidney Disease: A Cross-Sectional Study Based on a Health Check-Up Population, BMJ Open. (2022) 12, no. 12, 10.1136/bmjopen-2022-066243. PMC9806076 36581406

[5] Li Z. , Liu Q. , and Yao Z. , The Serum Uric Acid-To-High-Density Lipoprotein Cholesterol Ratio is a Predictor for All-Cause and Cardiovascular Disease Mortality: A Cross-Sectional Study, Frontiers in Endocrinology. (2024) 15, 10.3389/fendo.2024.1417485. PMC11427315 39345882

[6] Zhou X. and Xu J. , Association Between Serum Uric Acid-To-High-Density Lipoprotein Cholesterol Ratio and Insulin Resistance in Patients With Type 2 Diabetes Mellitus, Journal of Diabetes Investigation. (2024) 15, no. 1, 113–120, 10.1111/jdi.14086. 37737515 PMC10759725

[7] Xie Y. , Huang K. , Zhang X. et al., Association of Serum Uric Acid-To-High-Density Lipoprotein Cholesterol Ratio With Non-Alcoholic Fatty Liver Disease in American Adults: A Population-Based Analysis, Front Med-Lausanne. (2023) 10, 10.3389/fmed.2023.1164096. PMC10225665 37256087

[8] Cui Y. and Zhang W. , Long-Term Cardiovascular Risk and Mortality Associated With Uric Acid to HDL-C Ratio: A 20-Year Cohort Study in Adults Over 40, Scientific Reports. (2025) 15, no. 1, 10.1038/s41598-025-99205-3. PMC12022283 40275048

[9] Rubio-Ruiz M. E. , Guarner-Lans V. , Pérez-Torres I. , and Soto M. E. , Mechanisms Underlying Metabolic Syndrome-Related Sarcopenia and Possible Therapeutic Measures, International Journal of Molecular Sciences. (2019) 20, no. 3, 10.3390/ijms20030647, 2-s2.0-85061120975. PMC6387003 30717377

[10] Şenoymak İ. , Egici M. T. , and Şenoymak M. C. , Sarcopenia and Associated Factors in Adults Aged 40 and Above: A Study Conducted in Primary Healthcare, Cureus Journal of Medical Science. (2024) 16, no. 8, 10.7759/cureus.67618. PMC11416839 39310536

[11] Zhou S. , Wu L. , Si H. , and Shen B. , Longitudinal Association Between Uric Acid and Incident Sarcopenia, Nutrients. (2023) 15, no. 14, 10.3390/nu15143097. PMC10384494 37513515

[12] Lin Y. , Wang X. , Yao W. , Sun Y. , Zhou J. , and Feng F. , Causality Between Urate Levels With Sarcopenia-Related Traits: A Bi-Directional Mendelian Randomization Study, Frontiers in Endocrinology. (2023) 14, 10.3389/fendo.2023.1252968. PMC10637380 37955003

[13] Jiang Y. , Xu B. , Zhang K. et al., The Association of Lipid Metabolism and Sarcopenia Among Older Patients: A Cross-Sectional Study, Scientific Reports. (2023) 13, no. 1, 10.1038/s41598-023-44704-4. PMC10579328 37845303

[14] Cruz-Jentoft A. J. and Sayer A. A. , Sarcopenia, The Lancet. (2019) 393, no. 10191, 2636–2646, 10.1016/S0140-6736(19)31138-9, 2-s2.0-85067884022. 31171417

[15] Wang H. , Ba Y. , Gao X. et al., Association Between Serum Uric Acid to High Density Lipoprotein-Cholesterol Ratio and Arterial Stiffness in a Japanese Population, Medicine. (2023) 102, no. 31, 10.1097/MD.0000000000034182. PMC10402983 37543775

[16] Huang H. , Yu X. , Jiang S. et al., The Relationship Between Serum Lipid With Sarcopenia: Results From the NHANES 2011–2018 and Bidirectional Mendelian Randomization Study, Experimental Gerontology. (2024) 196, 10.1016/j.exger.2024.112560. 39214262

[17] Chung S. M. , Moon J. S. , and Chang M. C. , Prevalence of Sarcopenia and Its Association With Diabetes: A Meta-Analysis of Community-Dwelling Asian Population, Front Med-Lausanne. (2021) 8, 10.3389/fmed.2021.681232. PMC8174659 34095184

[18] Morley J. E. , Hormones and Sarcopenia, Current Pharmaceutical Design. (2017) 23, no. 30, 4484–4492, 10.2174/1381612823666161123150032, 2-s2.0-85032294583. 27881060

[19] Kalfaoglu M. E. , Could Serum Uric Acid to HDL Cholesterol Ratio Predict Sacroiliitis?, PLoS One. (2023) 18, no. 10, 10.1371/journal.pone.0289624. PMC10593233 37871023

[20] Sivritepe R. , Basat S. U. , Gökmen N. , Duygu A. , Siyer ÖK. , and Tiril S. M. , Could Urinary Amino Acids Be as New Biomarkers for Detection of Sarcopenia?, Bratislava Medical Journal. (2025) 126, no. 1, 41–53, 10.1007/s44411-024-00009-0.

[21] Gecegelen E. , Ucdal M. , Baş A. O. et al., Inflammation-Related Indices and Low Physical Performance in Older Adults With Unintentional Weight Loss: A Cross-Sectional Study, BMC Geriatrics. (2026) 26, no. 1, 10.1186/s12877-025-06960-z. PMC12879472 41526841

