# Supplementary material for: Association Between Uric Acid‐to‐HDL Cholesterol Ratio and Sarcopenia: Sex‐Specific Patterns From NHANES 2011–2018
Source: Int J Endocrinol. 2026 May 11;2026:9746996. doi: 10.1155/ije/9746996 (PMC13161460; doi:10.1155/ije/9746996)
Supplement: Supplementary file 1 — Supporting Information Table S1: Variance Inflation Factor Analysis for Predictor Variables in the Multivariable Model. Table S2: Boruta Feature Filtering Results with Multiple Variables. [file IJE-2026-9746996-s001.docx]

**Supplementary materials**

**Table S1** Variance Inflation Factor Analysis for Predictor Variables in the Multivariable Model

| **Variable** | **GVIF** | **Df** | **GVIF^(1/(2×Df))** |
| --- | --- | --- | --- |
| Hypertension | 1.2047 | 1 | 1.0976 |
| Diabetes | 1.1347 | 1 | 1.0652 |
| Smoking | 1.1467 | 1 | 1.0708 |
| Cancer | 1.0474 | 1 | 1.0234 |
| Sex | 1.3406 | 1 | 1.1578 |
| Age | 1.5125 | 1 | 1.2298 |
| Race | 1.3782 | 4 | 1.0409 |
| Education Level | 1.6458 | 4 | 1.0643 |
| Marital Status | 1.4538 | 5 | 1.0381 |
| PIR | 1.3984 | 1 | 1.1825 |
| UHR | 1.3053 | 1 | 1.1425 |
| Total Cholesterol | 1.0719 | 1 | 1.0353 |

**Note:** GVIF^(1/(2×Df)): Adjusted GVIF accounting for variable dimensionality. All variables demonstrated GVIF^(1/(2×Df)) values <1.5, indicating absence of substantial multicollinearity in the model.

**Abbreviations**: UHR= Uric Acid to High-Density Lipoprotein Cholesterol Ratio; PIR=Poverty-to-Income Ratio. GVIF = Generalized Variance Inflation Factor; Df = Degrees of freedom;

**Table S2** Boruta Feature Filtering Results with Multiple Variables

| **Variable Name** | **Mean Importance** | **Median Importance** | **Min Importance** | **Max Importance** | **Decision** |
| --- | --- | --- | --- | --- | --- |
| Race | 24.88 | 24.9653 | 22.4422 | 27.3912 | Confirmed |
| Age | 18.6388 | 18.3169 | 16.2366 | 21.7653 | Confirmed |
| Diabetes | 12.6657 | 12.9472 | 9.2725 | 15.9522 | Confirmed |
| Marital Status | 8.4625 | 8.3125 | 6.414 | 11.2389 | Confirmed |
| UHR | 8.2199 | 8.1596 | 6.2744 | 10.1243 | Confirmed |
| TC | 7.2537 | 7.5735 | 4.8706 | 8.8032 | Confirmed |
| Hypertension | 7.2263 | 7.5733 | 5.1906 | 9.8867 | Confirmed |
| Education Level | 7.1998 | 6.9223 | 5.6331 | 10.2796 | Confirmed |
| Sex | 4.8392 | 4.7141 | 3.0133 | 7.2783 | Confirmed |
| Smoking | 4.5182 | 4.287 | 2.89 | 6.7321 | Confirmed |
| Cancer | 0.3656 | 0.6337 | -2.6416 | 2.3266 | Rejected |

**Abbreviations:** UHR = Uric Acid to High-Density Lipoprotein Cholesterol Ratio;
